# Supplementary material for: Repression of developmental transcription factor networks triggers aging-associated gene expression in human glial progenitor cells
Source: Nat Commun. 2024 May 8;15:3873. doi: 10.1038/s41467-024-48118-2 (PMC11079006; doi:10.1038/s41467-024-48118-2)
Supplement: Supplementary file 1 — Supplementary Information [file 41467_2024_48118_MOESM1_ESM.pdf]

Repression of developmental transcription factor networks triggers aging-associated gene expression in human glial progenitor cells Mariani et al.

Description of Supplementary Figures

7 Supplementary Figures

Supplementary Figure 1. Fetal hGPC enrichment via CD140 selection. *Related to Figure 1*

Supplementary Figure 2. Shared motifs of active transcription factors in fetal or adult hGPCs. *Related to Figure 3*

Supplementary Figure 3. Adult repressor isoform expression. *Related to Figures 4 and 5*

Supplementary Figure 4. ESC- and iPSC-derived hGPCs resemble fetal hGPCs. *Related to Figures 4 and 5*

Supplementary Figure 5. Transcription factor regulation of miRNAs provides post-transcriptional modulation of glial aging gene expression. *Related to Figure 6*

Supplementary Figure 6. Glial Explorer: An intuitive Shiny app to query human glial transcriptional expression.

Supplementary Figure 7. Gating strategy for GPC FACS isolation.

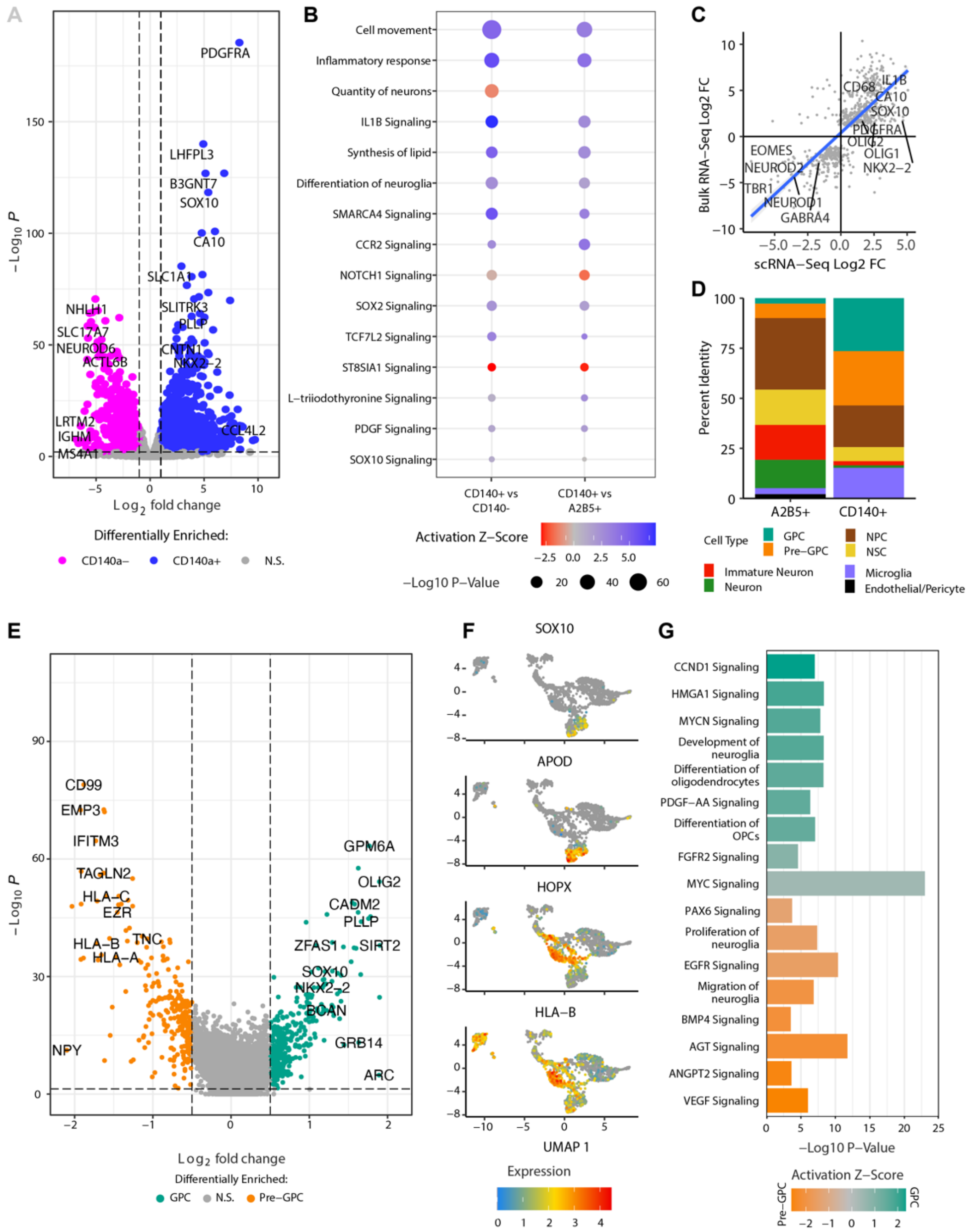

Supplementary Figure 1

Fetal hGPC enrichment via CD140a selection

A. Volcano plot of FACS selected CD140a$^+$ vs CD140a$^-$ VZ/SVZ fetal brain dissociates. **B.** Significant curated Ingenuity Pathway Analysis terms for both genesets (FDR <0.05, Log2FC > 1, calculated in DESeq2). Size represents $\log_{10}$ p-value and color represents activation Z-Score (*blue*, CD140a$^+$; *red*, A2B5$^+$ or CD140a$^-$). **C.** Scatter plot of differentially expressed bulk RNA-Seq $\log_2$ fold changes vs. pseudobulk $\log_2$ fold changes between the entire populations of CD140a$^+$ and A2B5$^+$ fetal hGPC isolates indicating comparability of the data obtained from bulk and single cell analyses. **D.** Stacked bar plot of cell type distributions in each sample. **E.** Volcano plot of GPC vs pre-GPC populations (FDR < 0.01, $\log_2$FC > 0.5, Seurat MAST test). **F.** Feature plots of curated genes differentially expressed between hGPCs and pre-hGPCs. **G.** Curated significantly-enriched GPC and pre-GPC IPA terms, indicating their -log10 p-value and activation Z-Score (FDR <0.001). Source data are provided as a Source Data file.

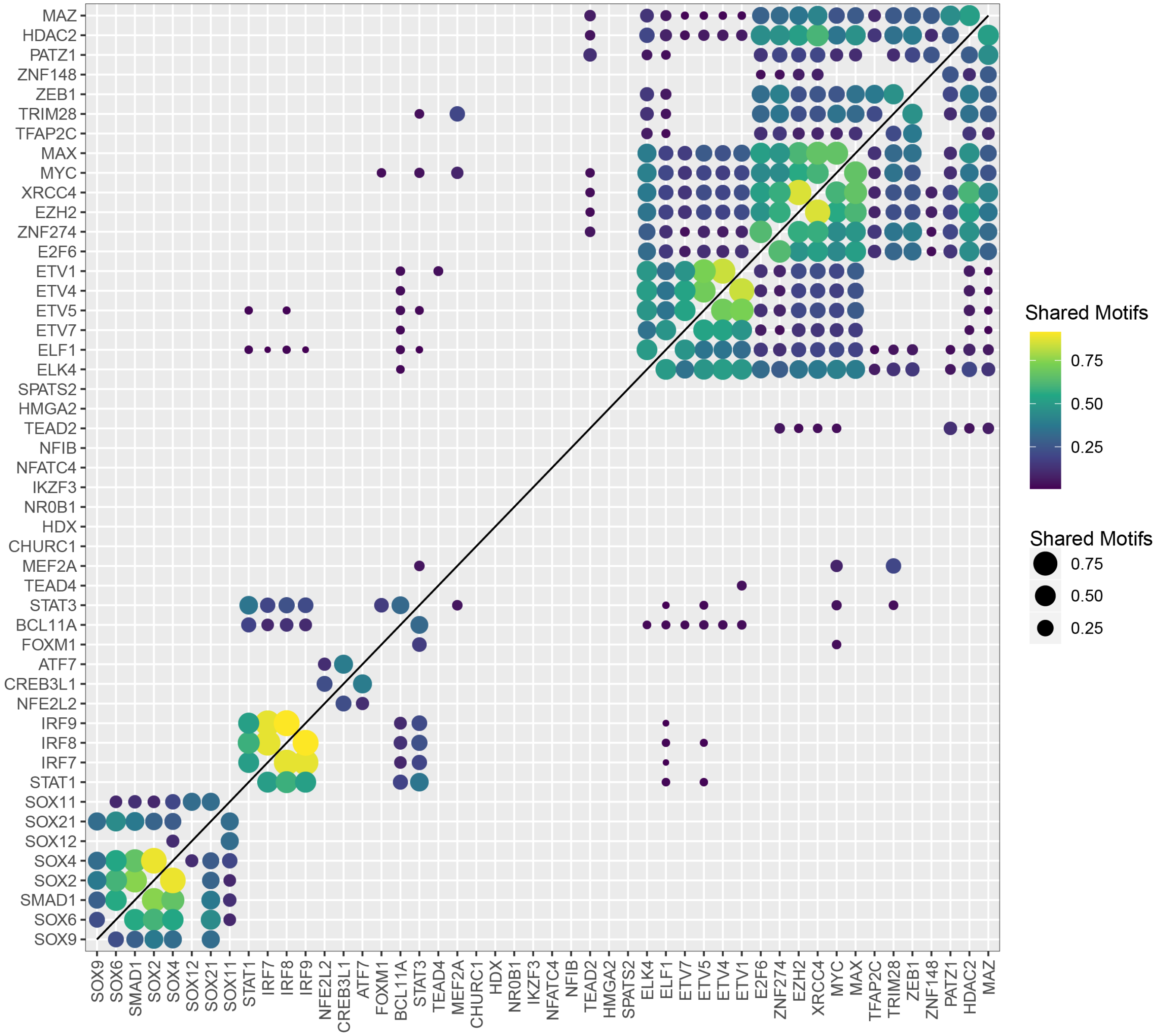

Supplementary Figure 2

Shared motifs of active transcription factors in fetal and adult hGPCs

Matrix of similarity between all predicted (Normalized Enrichment Score > 3) active transcription factors in fetal and adult GPCs. Similarity was calculated in igraph. Size and color indicate degree of motifs that are shared between transcription factors. Source data are provided as a Source Data file.

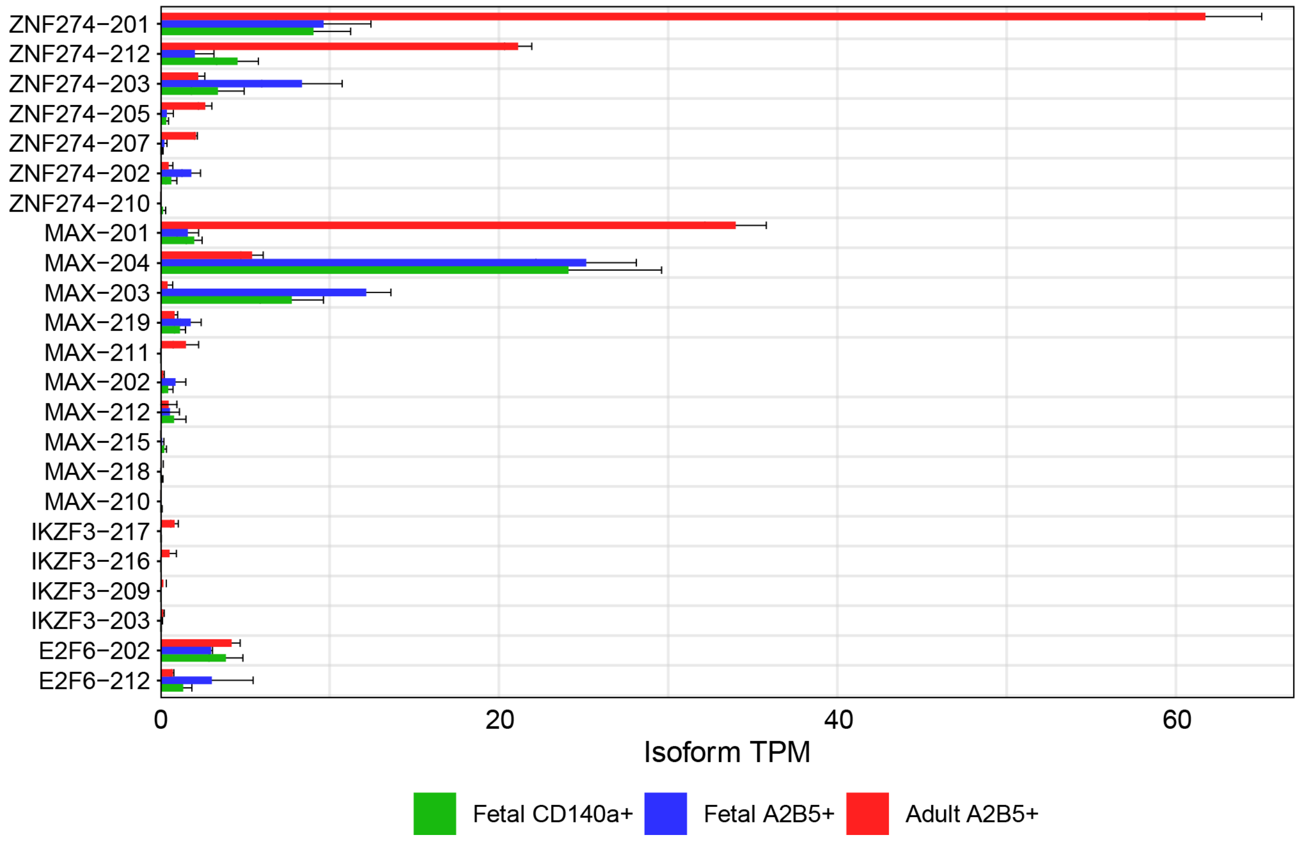

Supplementary Figure 3
Adult repressor isoform expression

Bar plots of transcripts per million (TPMs) of the major protein coding isoforms of selected adult repressors (see **Fig. 4**), as identified in each hGPC group. Source data are provided as a Source Data file. Error bars ± SEM.

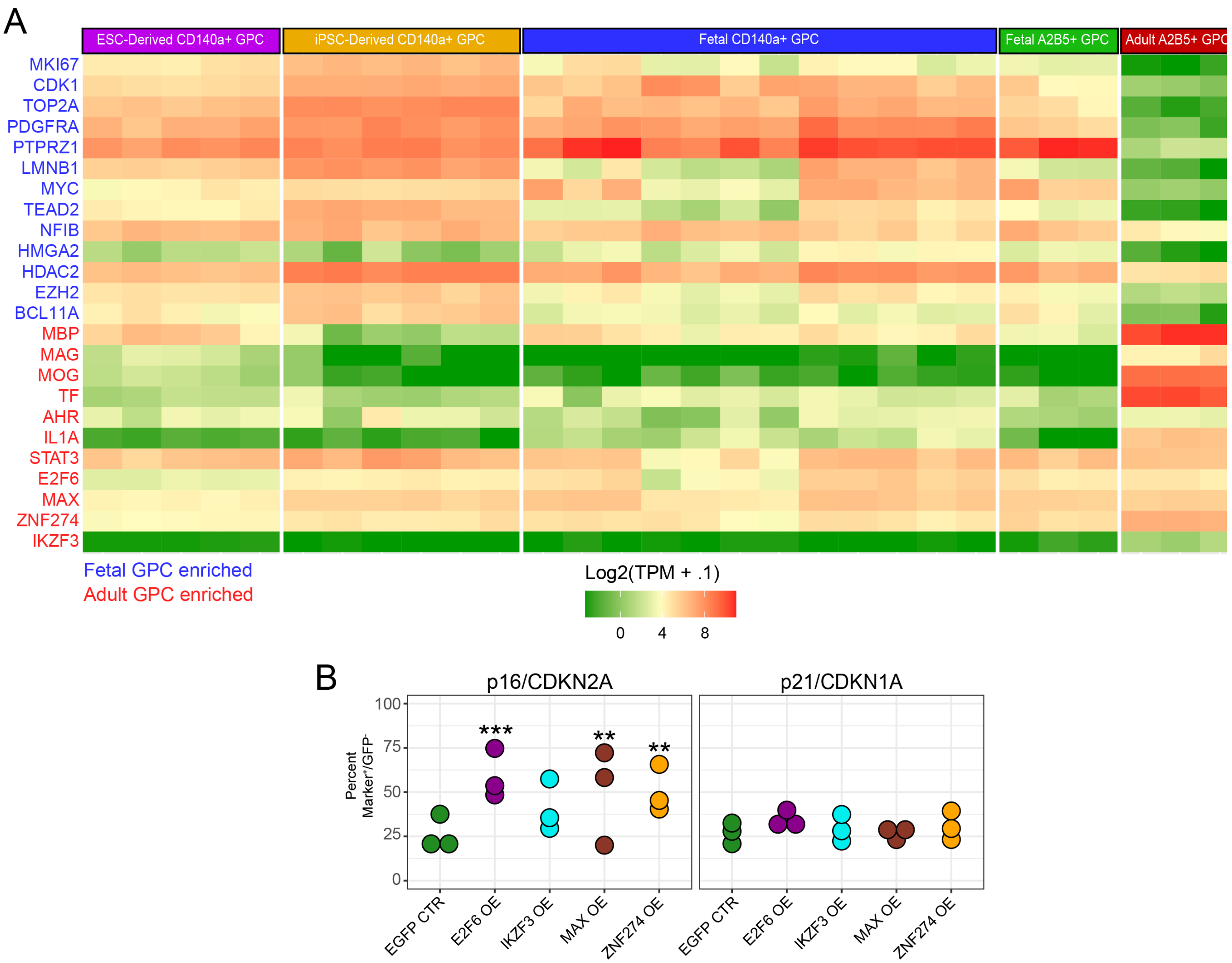

Supplementary Figure 4

ESC- or iPSC-derived hGPCs resemble fetal hGPCs

A. hESC- (WA09) or iPSC-derived (C27) hGPCs were isolated via CD140a$^+$ FACS and assayed via bulk RNA-sequencing for comparison to primary fetal and adult hGPCs (n=5 WA09, n=6 C27, n=12 CD140a+ Fetal, n=3 A2B5+ Fetal, n=3 A2B5+ Adult hGPCs). Abundance of relevant glial age-associated genes, including those in our active transcription factor cohort, are displayed as $\log_2$ (transcripts per million + .1). **B.** P16 and P21 immunostaining of EGFP CTR or OE virus infected WA09 hGPCs 7 days following doxycycline treatment in EGFP$^-$ hGPCs (n=3 biologically independent samples). Post hoc pairwise comparisons of overexpression conditions to EGFP controls in **B** were calculated via estimated marginal means tests of linear models following regression of a cell batch effect.

FDR adjusted p-values: *<0.05, ** <0.01, *** <0.001. Source data are provided as a Source Data file; exact p values are listed therein. Error bars ± SEM.

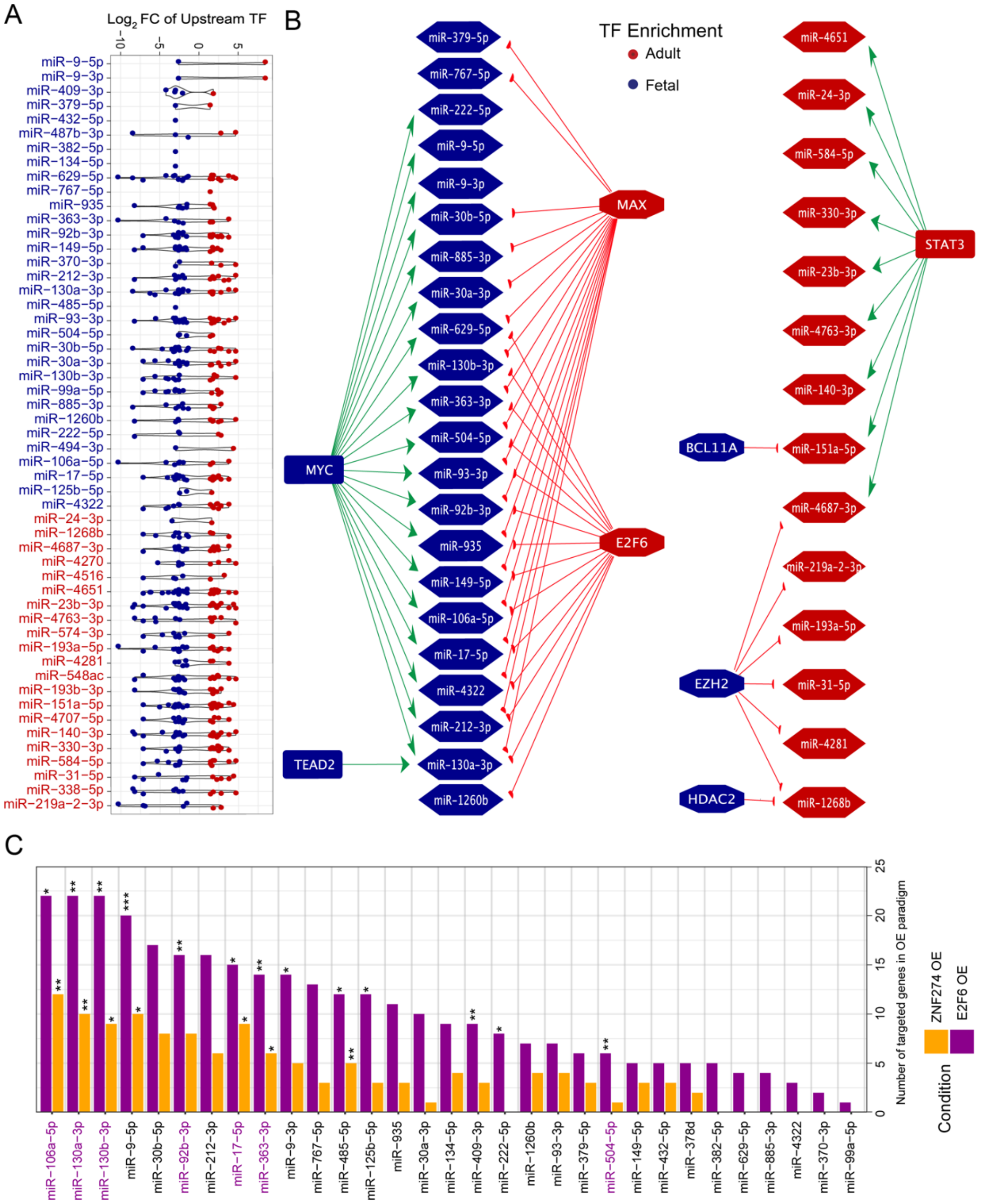

Supplementary Figure 5

Transcription factor regulation of miRNAs provides post-transcriptional modulation of glial aging

A. Log2 FC violin plots of significant (FDR < 0.01) adult vs fetal hGPC transcription factors predicted (transmiR) to be upstream of differentially expressed adult vs fetal GPC miRNAs (**Fig. 6**). **B.** Network of identified transcription factors from **Figure 3** and their predicted regulation of differentially expressed adult vs fetal hGPC miRNAs. **C.** Number of predicted direct miRNA targets of fetal-enriched miRNAs in E2F6 and ZNF274 OE C27 hGPCs vs EGFP CTR hGPCs (**Figure 5**). Significant enrichment of these targets was calculated using Fisher Exact tests of significantly repressed genes vs. activated in each geneset. FDR adjusted p-values: *<0.05, ** <0.01, *** <0.001. Source data are provided as a Source Data file; exact p values are listed there.

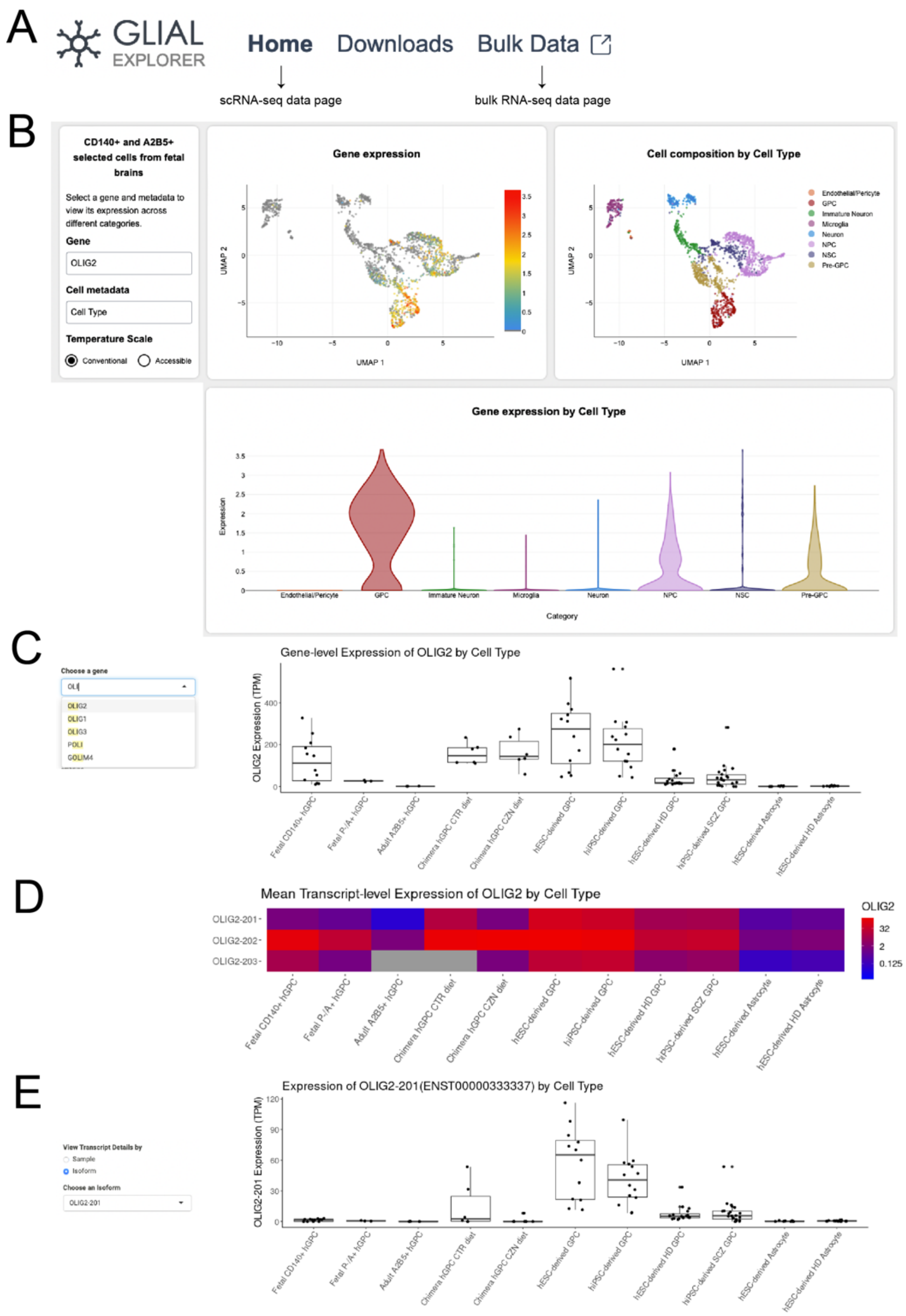

Supplementary Figure 6

Glial Explorer: an intuitive Shiny app to query human glial transcriptional expression

A. The landing page of glialexplorer.io includes links to both our scRNA-seq data page and bulk RNA-seq data page. **B.** scRNA-Seq data can be explored by querying a gene, and viewing feature plots of their expression, or violin plots. Cell composition by meta data may also be explored. **C.** To use the human glial bulk RNA-Seq database, first select the gene of interest from the dropdown menu. Plots of gene abundance as transcripts per million (TPM) are graphed by glial cell type and condition. **D.** Exploration of splice variants and their relative abundances are displayed for each queried gene. **E.** Bar plots of splice variant abundances may be viewed across all datasets; alternatively, a single data set can be viewed to examine the heterogeneity of its isoforms.

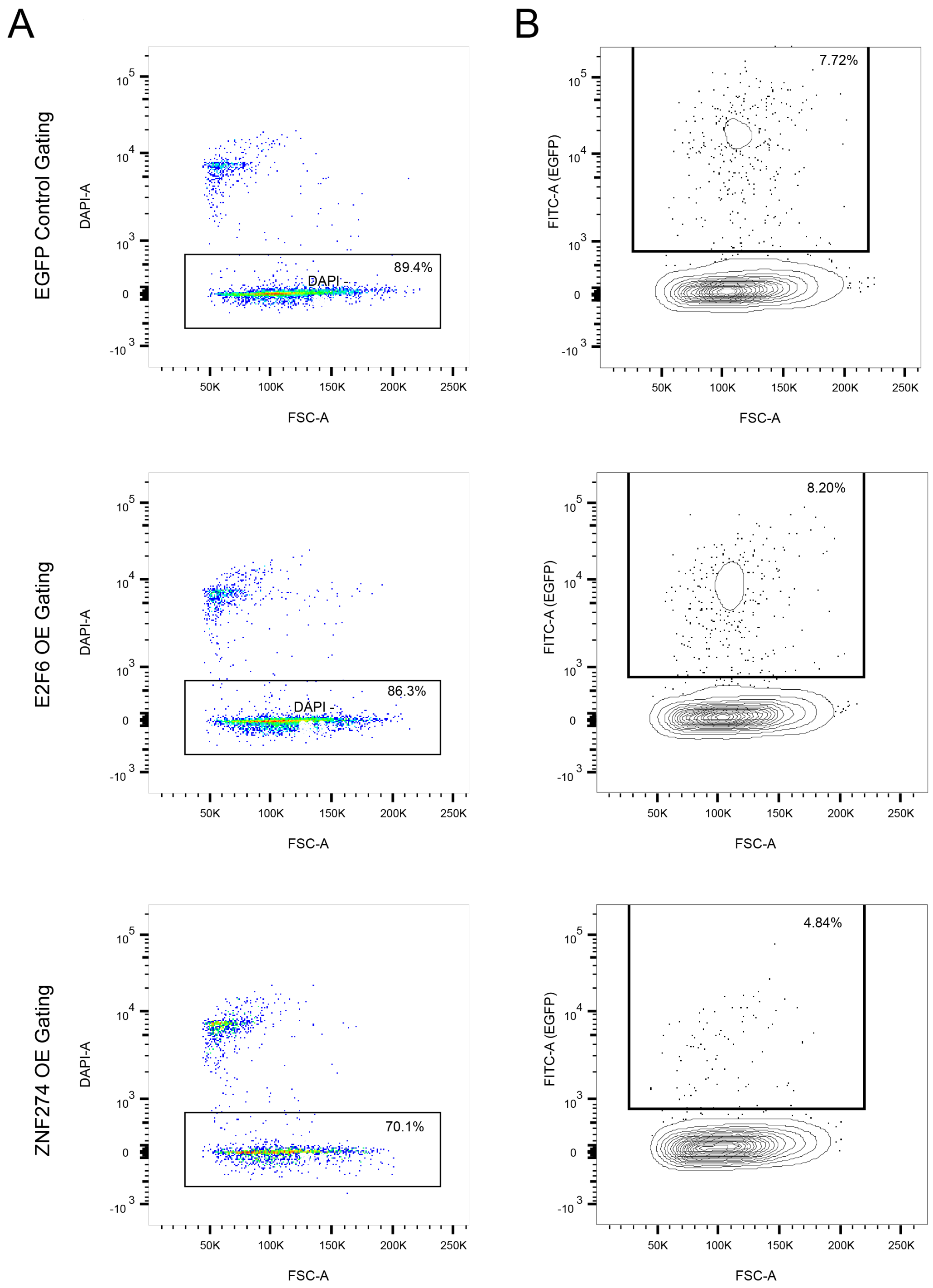

Supplementary Figure 7

Gating strategy for GPC FACS isolation

A. ESC- or iPSC-derived hGPCs were first selected for live cells based on DAPI negativity. **B.** Infected live hGPCs were isolated based on EGFP signal.
