# Supplementary material for: Repression of developmental transcription factor networks triggers aging-associated gene expression in human glial progenitor cells
Source: Nat Commun. 2024 May 8;15:3873. doi: 10.1038/s41467-024-48118-2 (PMC11079006; doi:10.1038/s41467-024-48118-2)
Supplement: Supplementary file 3 — Description of Additional Supplementary Files [file 41467_2024_48118_MOESM3_ESM.pdf]

Repression of developmental transcription factor networks triggers aging-associated gene expression in human glial progenitor cells Mariani et al.

Description of Additional Supplementary Files

7 Supplementary Data

Supplementary Data 1. Differentially expressed gene and significant IPA terms between fetal hGPCs. *Related to Figure 1 and Supplementary Figure 1.*

Supplementary Data 2. Significant scRNA-seq population markers and fetal GPC vs Pre-GPC differentially expressed genes, significant IPA terms, and significant transcription factor regulons. *Related to Figure 1.*

Supplementary Data 3. Differentially expressed genes, significant IPA terms, and predicted active transcription factors between adult and fetal GPCs. *Related to Figures 2 and 3*

Supplementary Data 4. Differentially expressed gene and significant IPA terms of hGPCs transduced to over-express E2F6 or ZNF274, vs an EGFP control. *Related to Figure 5.*

Supplementary Data 5. Differentially expressed miRNAs, predicted miRNA targets and transcription factor regulators of adult vs fetal hGPC miRNAs. *Related to Figure 6 and Supplementary Figure 5.*

Supplementary Data 6. qPCR primer sequences

Supplementary Data 7. Antibody information

Supplementary Data *(A-D refer to sequential tabs on each Excel spreadsheet)*

Supplementary Data 1

Differentially expressed gene and significant IPA terms between fetal hGPCs *Related to Figure 1 and Supplementary Figure 1.*

A. Differentially expressed (FDR < 0.01, Log2FC > 1, DESeq2) genes between CD140a$^+$ and A2B5$^+$/PSA-NCAM$^-$ hGPCs. **B)** Genes differentially expressed (FDR <0.01, Log2FC>1, DESeq2) between CD140$^+$ and CD140$^-$ fetal VZ/SVZ cells. **C.** Significant IPA terms (FDR < 0.05) of the genelist in **A**. **D.** Significant IPA terms (FDR < 0.05) of the genelist in **B**.

Supplementary Data 2

Significant scRNA-seq population markers, and fetal GPC vs pre-GPC differentially expressed genes, IPA terms, and transcription factor regulons *Related to Figure 2.*

A. Significant cell type markers (FDR < 0.05, Seurat MAST test). **B.** Genes differentially expressed between GPCs and pre-GPCs (FDR <0.01, Log_2FC > 0.5, Seurat MAST test), and **C)** their significant IPA terms (FDR <0.001). **D.** Significant differential activation of SCENIC derived transcriptional regulons (FDR <0.01, Seurat Wilcoxon sum-rank test) between GPCs and Pre-GPCs.

Supplementary Data 3

Differentially expressed genes, IPA terms, and predicted active transcription factors between adult and fetal GPCs *Related to Figures 3-4 and Supplementary Figure 4.*

A. Significant differentially expressed genes between either CD140$^+$ or PSA-NCAM$^-$/A2B5$^+$ Fetal GPCs and A2B5$^+$ Adult GPCs (FDR < 0.01, Log2FC > 1, DESeq2). **B.** Significant IPA terms between the intersection of fetal CD140$^+$ vs. adult A2B5$^+$ GPCs and Fetal PSA-NCAM$^-$/A2B5$^+$ vs Adult A2B5$^+$ GPCs (FDR < 0.001). **C.** Active transcriptional regulons as determined by RcisTarget on the geneset of **A,** and their predicted differentially expressed targets (NES > 3).

Supplementary Data 4

Differentially expressed gene and significant IPA terms of hIPSC-derived human GPC cultures overexpressing either E2F6 or ZNF274 vs an EGFP control *Related to Figure 5.*

A. Significant GPC Markers (FDR < 0.05, Seurat MAST test). **B-C.** Differential expression of genes in E2F6 over-expression **(B)** or ZNF274 over-expression **(C)** conditions, vs EGFP Ctr (FDR <0.05, Log_2FC >0.25, Seurat MAST test). **D,** Significant IPA terms of E2F6 or ZNF274 overexpression, both vs. **E.** EGFP Ctr (FDR <0.05). **F-G,** Active transcriptional regulons as determined by RcisTarget on the E2F6 **(F)** or ZNF274 **(G)** genesets, and their predicted differentially expressed targets (NES > 3).

Supplementary Data 5

Differentially expressed miRNAs, predicted miRNA targets, and predicted transcription factor regulators of adult vs fetal hGPC miRNAs *Related to Figure 6 and Supplementary Figure 7.*

A. Differentially expressed miRNAs between fetal CD140$^+$ and adult A2B5$^+$ hGPCs (FDR < 0.01, calculated in limma). **B.** Predicted differentially expressed targets from **Supplementary Data 3A,** of differentially expressed miRNAs in **A** determined via miRNAtap. **C.** TransmiR predicted adult vs fetal GPC differentially expressed miRNA regulation via transcriptional regulators in **Figure 4**.

Supplementary Data 6. qPCR Primer sequences.

Supplementary Data 7. Antibody information.
